# Enhanced recovery after surgery in cytoreductive surgery and hyperthermic intraperitoneal chemotherapy: national survey of peri-operative practice by Indian society of peritoneal surface malignancies

**DOI:** 10.1515/pp-2022-0198

**Published:** 2023-05-22

**Authors:** Sampige Prasanna Somashekhar, Suryanarayana Deo, Subramanyeshwar Rao Thammineedi, Harit Chaturvedi, Ganesh Mandakukutur Subramanya, Rama Joshi, Jagdish Kothari, Ayyappan Srinivasan, Kumar C. Rohit, Mukurdipi Ray, Bharat Prajapati, Hemanth Guddahatty Nanjappa, Rajagopalan Ramalingam, Aaron Fernandes, Kyatsandra Rajagopal Ashwin

**Affiliations:** Aster International Institute of Oncology , Aster hospital , Bengaluru, India; Surgical Oncology, Basavatarakam Indo-American Cancer Hospital and Research Institute, Hyderabad, India; Max Institute of Cancer care, New Delhi, India; Vydehi Institute of Medical Sciences and Research Centre, Bengaluru, India; Gynaecological Oncology, Fortis Memorial Research Institute, Gurgaon, New Delhi, India; HCG Cancer Centre Ahmedabad, Ahmedabad, India; Apollo Hospitals, Chennai, India; Department of Surgical Oncology, All India Institute of Medical Sciences, New Delhi, India

**Keywords:** cytoreductive surgery (CRS), enhanced recovery after surgery (ERAS), hyperthermic intraperitoneal chemotherapy (HIPEC), peritoneal surface malignancy (PSM)

## Abstract

**Objectives:**

The Enhanced recovery after surgery (ERAS) program is designed to achieve faster recovery by maintaining pre-operative organ function and reducing stress response following surgery. A two part ERAS guidelines specific for Cytoreductive surgery (CRS) and Hyperthermic Intraperitoneal Chemotherapy (HIPEC) was recently published with intent of extending the benefit to patients with peritoneal surface malignancies. This survey was performed to examine clinicians’ knowledge, practice and obstacles about ERAS implementation in patients undergoing CRS and HIPEC.

**Methods:**

Requests to participate in survey of ERAS practices were sent to 238 members of Indian Society of Peritoneal Surface malignancies (ISPSM) via email. They were requested to answer a 37-item questionnaire on elements of preoperative (n=7), intraoperative (n=10) and postoperative (n=11) practices. It also queried demographic information and individual attitudes to ERAS.

**Results:**

Data from 164 respondents were analysed. 27.4 % were aware of the formal ERAS protocol for CRS and HIPEC. 88.4 % of respondents reported implementing ERAS practices for CRS and HIPEC either, completely (20.7 %) or partially (67.7 %). The adherence to the protocol among the respondents were as follows: pre operative (55.5–97.6 %), intra operative (32.6–84.8 %) and post operative (25.6–89 %). While most respondents considered implementation of ERAS for CRS and HIPEC in the present format, 34.1 % felt certain aspects of perioperative practice have potential for improvement. The main barriers to implementation were difficulty in adhering to all elements (65.2 %), insufficient evidence to apply in clinical practice (32.4 %), safety concerns (50.6 %) and administrative issues (47.6 %).

**Conclusions:**

Majority agreed the implementation of ERAS guidelines is beneficial but are followed by HIPEC centres partially. Efforts are required to overcome barriers like improving certain aspects of perioperative practice to increase the adherence, confirming the benefit and safety of protocol with level I evidence and solving administrative issues by setting up dedicated multi-disciplinary ERAS teams.

## Introduction

The multimodality treatment of cytoreductive surgery (CRS) and heated intraperitoneal chemotherapy (HIPEC) combines radical surgery with circulation of heated chemotherapy in the peritoneal cavity for selected patients with Peritoneal surface malignancies (PSM) [1].

This radical surgery induces metabolic and inflammatory responses, associated with higher morbidity, prolonged in-patient stay and longer recovery compared to other gastrointestinal and gynaecological oncological procedures. Enhanced recovery after surgery (ERAS) protocols represent fundamental shifts in surgical practice designed to achieve early recovery by maintaining preoperative organ function and reducing the profound stress response following surgery [2], [3], [4]. A survey showed CRS and HIPEC has been well accepted by the oncological community [5].

ERAS programs offer a practical, evidence based, patient-centric practices to eliminate ambiguities, disparities to achieve best surgical care. There is already significant evidence indicating that ERAS protocols leads to improved outcomes in major abdominal and extra-abdominal surgical procedures, while being safe [5], [6], [7], [8], [9], [10], [11], [12], [13].

There is a wide acceptance to ERAS in western countries, whereas in the developing countries, ERAS programs are still facing considerable challenges for application [14]. Incorporation of pre-, intra-, and postoperative practices of the ERAS pathway in the management of patients undergoing CRS-HIPEC causes synergistic effect of early reversal of the pathophysiological responses and thereby hasten recovery and reduce complications. Preliminary experience of partial application of ERAS in CRS and HIPEC patients showed a reduction in overall intravenous fluids, postoperative narcotic use, complication rates [15], [16], [17].

Recently Hubner et al. published the formal ERAS guidelines specific to CRS and HIPEC with several key elements of including preoperative counselling, optimization of nutrition, standardized analgesic and aesthetic regimens, early mobilization and special consideration for HIPEC [18, 19]. This formal ERAS protocol represents a significant change in practice for CRS and HIPEC, but also poses a challenge for adherence and compliance. It is unclear to what degree ERAS guidelines are implemented in HIPEC centres of excellence.

The aim of this study was to evaluate utilization of ERAS in CRS and HIPEC, assess knowledge, current practices and barriers to adherence.

## Methods

The survey was submitted to all the members of Indian society of peritoneal surface malignancies (ISPSM) via email, as extracted from the membership directory of the association. Reminders were sent to the non-responders via e-mail. The questionnaire was developed after extensive research for various elements of the ERAS protocol. The questionnaire was pilot-tested among the ERAS specialists and oncologists within our institution for assessment and changes were made based on feedback.

**Figure 1:**
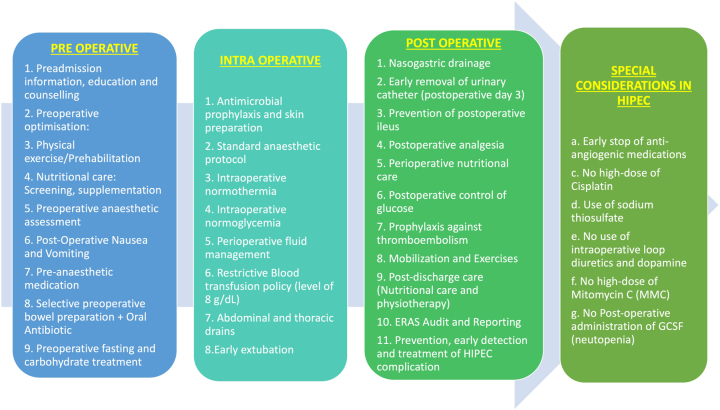
Components of ERAS for CRS and HIPEC.

The questionnaire had 37 items and was divided into five sections. Section one dealt with the demographics and their awareness of ERAS protocols. The second, third and fourth sections consisted of questions investigating elements of preoperative (n=7), intraoperative (n=10) and postoperative (n=11) practices and their extent of implementation. Non-essential components of the protocol were not included in the questionnaire. The last part assessed the individual attitudes and barriers for implementation and adherence Figure 1.

A descriptive statistical analysis was carried out and described quantitative and qualitative data according to means (± standard deviation), medians (range) and percentages. The percentages were calculated over all the responses received for each question. Percentages and frequencies were used for the descriptive analysis of the data.

## Results

**Figure 2:**
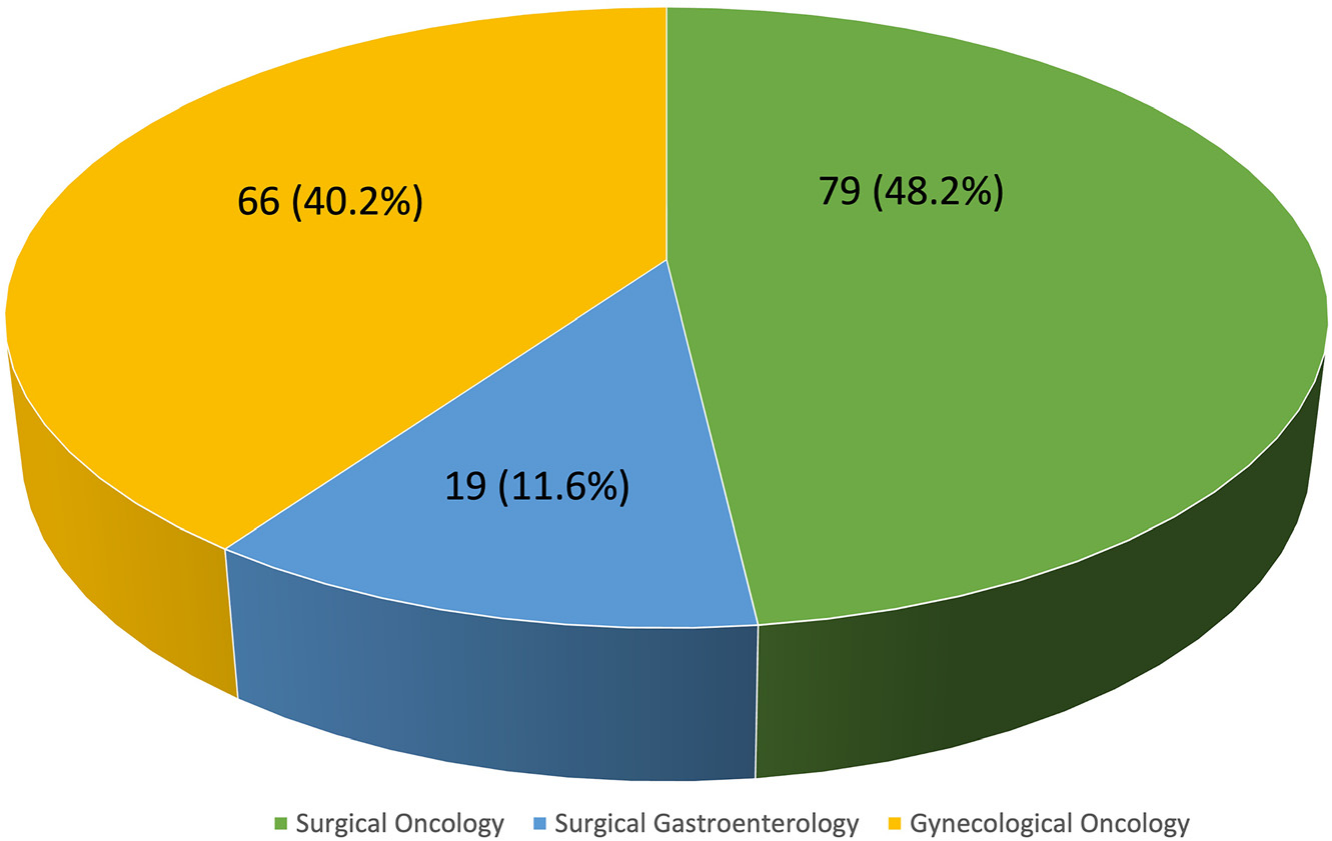
Subspecialty of the survey respondents.

Out of 238 active surgical members of ISPSM who were contacted for the survey, 164 (68.9 %) replied with completed questionnaire. We compiled and analysed the results from the participants (Figure 2).

Demographic characteristics of survey respondents are shown in Table 1. Our nation has a huge variation in the types of institutes delivering healthcare in our country, 23.1 % of the doctors practice in a public medical research university hospitals while rest are involved in private care. Although not officially registered with ERAS society 95.7 % were already implementing ERAS for other surgical specialities. Eighty-eight percent of respondents reported implementing ERAS practices for CRS and HIPEC, completely (20.7 %) or partially (67.7 %).

**Table 1:** Demographic characteristics of survey respondents.

Sl	Parameters	n=164	%
1	What is the type of hospital where you practice? – Medical college – Private teaching institution – Private hospital	38 67 59	23.1 40.9 35.0
2	What is your specialty? – Surgical oncology – Surgical gastroenterology – Gynaecologic oncology	79 19 66	48.2 11.6 40.2
3	What’s your experience in performing CRS + HIPEC? – <2 years – 2–5 years – >5 years	26 70 68	15.9 42.7 41.4
4	Are you aware of dedicated guidelines of enhanced recovery after surgery (ERAS) for CRS + HIPEC? – Yes – No	45 129	27.4 72.6
5	Do you follow ERAS for CRS + HIPEC? – Yes – Partially – No	34 111 19	20.7 67.7 11.6
6	Peri-operative care protocols implemented in your institution for CRS HIPEC are based on guidelines set by? – MDT experts based on extrapolation – ERAS recommendations	130 34	79.3 20.7
7	What is the benefit of implementation of ERAS protocol in CRS HIPEC according to you ? (multiple choices possible) – Multimodal rehabilitation and reduce complications – Clinical research – Reduce costs – Reduce hospital stay – Not sure	137 61 29 112 29	83.4 37.2 17.7 68.3 17.7
8	Are you registered with ERAS society with dedicated ERAS co-ordinator? (Do you follow ERAS audit and reporting?) – Yes – No	12 152	7.3 92.7
9	Does your department offer ERAS for other surgical specialities? – Yes – Partially – No	41 116 7	25 70.7 4.3

Questionnaire responses for pre-operative and intra operative components of ERAS for CRS and HIPEC guidelines are shown in Tables 2 and 3. Routine bowel preparation with oral antibiotics was reported by 62.2 % of respondents. Pre-operative fasting for solids up to 8 h before surgery was reported by nearly 55.5 % of respondents; 18.3 % of respondents said they allowed clear liquids up to 2 h before surgery, 58 % 2–6 h before surgery, and only 26.2 % reported carbohydrate loading too. Pre-operative and intra-operative deep vein thrombosis prophylaxis was administered by more than 80 % of respondents. Low molecular weight heparin was the most common modality used for this purpose (71.3 %), while rest of respondents reported using stockings or pneumatic compression devices. In the anaesthetic considerations of the protocol total intravenous anaesthesia (59.1 %) with protective mechanical ventilation (71 %) was most commonly followed while invasive cardiac monitoring (32.6 %) was not routine. Goal-directed fluid therapy was reported by only 60.4 %. Regional anaesthesia was employed by most respondents (84.8 %) with epidural analgesia (59.1 %) being most popular followed by Transversus abdominis plane (TAP) block in 16.5 % followed by NSAIDs or Paracetamol (15.2 %). More than 50 % preferred to place drains routinely in all cases.

**Table 2:** Questionnaire responses for pre-operative components of ERAS guidelines for CRS and HIPEC.

Sl	Parameters	n=164	%
1	Preadmission information, education and counselling – Yes – No	160 4	97.6 2.4
2	Preoperative optimisation: Alcohol and smoking cessation – 4 weeks – <4 weeks	21 143	12.8 87.2
3	Do you perform prehabilitation and nutritional care – Yes – No	103 61	62.8 37.2
4	Do you give pre-operative antiemetic drugs for post-operative nausea and vomiting (PONV): – Yes – No – Not sure	94 19 51	57.3 11.6 31.1
5	Preoperative bowel preparation + oral antibiotic Always Selective	102 62	62.2 37.8
6	What is the preoperative fasting protocol in your institute? – 2 h for clear fluids, 6 h for solids – 2 h for clear fluids, 6 h for solids with carbohydrate loading – Nil per oral since midnight	30 43 91	18.3 26.2 55.5
7	Pre-operative DVT prophylaxis Unfractionated heparin Low molecular weight heparin Stockings Pneumatic compression device No	0 117 11 4 32	0 71.3 6.7 2.5 19.5

**Table 3:** Questionnaire responses for intra-operative components of ERAS guidelines for CRS and HIPEC.

Sl	Parameters	n=164	%
1	What type of general anaesthesia is usually included in the protocol in your hospital? Total intravenous anaesthesia with propofol Inhalational agents based Not definite	67 38 29	59.1 23.2 17.7
2	Do you use routine protective mechanical ventilation? Yes No Not sure	118 0 46	71 0 28
3	Do you use cardiac output monitoring routinely? Yes No	37 127	32.6 67.4
4	Do you use any modalities to measure and maintain intraoperative normothermia? Yes No	164 0	100 0
5	Do you perform goal-directed fluid management during surgery? Yes, goal-directed therapy protocol Not sure, at discretion of anaesthesia team No	99 58 7	60.4 35.4 4.2
6	Do you perform restrictive blood transfusion policy (threshold level of 8 g/dL) Yes No	71 93	43.3 56.7
7	Do you use regional analgesia? Epidural Spinal adjunct Nerve blocks (erector spinae/transversus abdominis block) No	97 15 27 25	59.1 9.1 16.5 15.3
8	Do you place abdominal and thoracic drains? Always Sometimes (bowel resection, splenectomy, bladder repair) No	85 65 14	51.8 39.6 8.6
9	Do you attempt for early extubation regularly? Yes No Not sure	70 55 39	62.7 13.5 23.8
10	Do you avoid use of postoperative antibiotic prophylaxis? Yes No Not sure	28 89 47	17.1 54.3 28.4

Table 4 shows the responses for the post-operative components of the guidelines. Nasogastric tube usage was common, reported to be used ‘always’ and ‘sometimes’ by 45.7 and 35.4 % respectively. Majority of respondents attempted for early indwelling catheter removal (78 %). Among strategies to hasten the return of bowel activity, chewing gum (64 %) and laxatives (29.9 %) were used commonly. Nearly 36 % of respondents indicated that they did not routinely employ substances to prevent post-operative ileus. Early post-operative diet initiation was not adhered by nearly three-fourth of the respondents, with clinical exam being the main indicator for resumption of diet. Fluid overload avoidance and monitoring was not done regularly by the clinicians (73.2 %). Patients were ambulated on the day of surgery by 10.4 % of respondents, while 63.4 % reported that patients typically ambulated on the first post-operative day.

**Table 4:** Questionnaire responses for post-operative components of ERAS guidelines for CRS and HIPEC.

Sl	Parameters	n=164	%
1	Do you avoid use of post-operative nasogastric drainage? Yes Sometimes (bowel resection, lesser omentectomy) No	31 58 75	18.9 35.4 45.7
2	Do you follow early removal of urinary catheter (<post op day 3) Yes Sometimes No	87 41 36	53 25 22
3	Which postoperative strategies do you use to accelerate the recovery of gastrointestinal function? (Choose all that apply) Chewing gum/Coffee Mu-opioid receptor antagonists Laxatives Prokinetics: Erythromycin Not routinely	105 18 49 11 59	64 11 29.9 6.7 36
4	Which method of regional analgesia do you prefer for postoperative pain control? Epidural analgesia Abdominal blocks Combination analgesia (paracetamol, NSAIDs) Opioids	88 25 42 9	53.6 15.3 25.6 5.5
5	When do you initiate post-operative regular diet? On POD 1 >POD 2 Based on clinical exam (bowel sounds/Passing of flatus)	19 23 122	11.6 14 74.4
6	Do you use pre-emptive parenteral nutrition? Yes No	146 18	89 11
7	Do you actively monitor and avoid post-operative fluid overload? Yes No Not sure	44 68 52	26.8 41.5 31.7
8	Prophylaxis against thromboembolism Till mobilization Till hospitalization 1 month	25 111 28	15.2 67.7 17.1
9	When do you start post-operative ambulation (average start time) Day of surgery POD1 >POD2	17 43 104	10.4 63.4 26.2
10	What is the duration of post operative physical exercises (>POD 2) 6 h <6 h Physiotherapist discretion	7 36 121	4.3 22 73.7
11	Do you follow strategies for prevention, early detection and treatment of HIPEC complication Early stop of anti-angiogenic medications No high-dose of cisplatin Use of sodium thiosulfate No use of intraoperative loop diuretics and dopamine No post-operative administration of GCSF (neutopenia)	160 129 31 48 129	97.5 78.6 18.9 29.3 78.6

### Attitudes to ERAS in CRS and HIPEC

65.9 % of clinicians responded that they would implement the published ERAS protocol in patients undergoing CRS and HIPEC without modifications. Overall, 65.2 % felt that ERAS protocols is a useful tool but few elements are difficult to adhere to. The other issues of concern for full implementation were safety (50.6 %), lack of evidence (35.4 %) and administrative issues (47.6 %). 84 % felt that ERAS protocols decreased hospital stays and 62.8 % re-admission rates. ERAS practices improved overall patients’ satisfaction according to 40.2 % of respondents, and 54.3 % felt that ERAS pathways improved patient outcomes. The surgeons were asked to evaluate the components of ERAS that are most effective interventions in improving the outcomes and responses were Intraoperative goal directed therapy (82.9 %), perioperative feeding practices (78 %) and early aggressive mobilisation (73.1 %). According to the respondents the elements that are difficult to implement included avoidance of mechanical bowel preparation (58 %), avoidance of drains (81.7 %), avoidance of post op fluid overload (48.1 %), regular diet initiation (73.2 %) Table 5.

**Table 5:** Respondents’ attitudes towards ERAS practices.

Sl	Parameters	n=164	%
1	Would you implement the published ERAS protocol in all your patients undergoing CRS + HIPEC without modifications? Yes No	108 56	65.9 34.1
2	Barriers of implementing ERAS in CRS + HIPEC (choose all that apply) Beneficial but difficult to adhere to all elements Evidence to support is insufficient Safety concerns Administrative issues	107 58 83 78	65.2 35.4 50.6 47.6
3	What are the benefits of implementing ERAS for CRS + HIPEC (choose all that apply) Improves patient outcome: Hospital stay and ICU stay Reduces re-admission rates Reduces complication Improves patient satisfaction	138 103 89 66	84.1 62.8 54.3 40.2
4	What are the interventions which make the current ERAS protocol for CRS HIPEC effective? (Choose all that apply) Early removal of indwelling catheter Intra operative goal directed therapy Short NPO duration, carbohydrate loading, early oral feeding and nutritional management Early aggressive mobilisation Ileus prevention strategies Epidural and postoperative pain management	91 136 128 120 39 85	55.5 82.9 78 73.1 23.8 51.8
5	What are the elements of ERAS guidelines that are difficult to implement? (Choose all that apply) Mechanical bowel preparation avoidance Oral regular diet initiation on POD1 Avoidance of post-operative antibiotics Avoidance of post-operative fluid overload Avoidance of naso gastric tubes Avoidance of intraperitoneal drains	95 120 108 79 88 134	58 73.2 65.8 48.1 53.6 81.7

## Discussion

With the availability of the formal ERAS CRS-HIPEC guidelines, we wanted to characterise the prevailing pattern of perioperative practice among specialist surgeons performing CRS andHIPEC. This is the first survey among HIPEC super specialists that has examined the degree of ERAS implementation and assess the knowledge attitudes and practice patterns based on published formal protocol. Previous surveys were based on a working ERAS guidelines which was extrapolated information from colorectal and gynaecological ERAS practices. Our survey revealed that ERAS for CRS and HIPEC was more widely adopted by surgeons with institutions already having dedicated ERAS program.

Despite majority already implementing ERAS protocol completely or partially among other surgical branches, we found that there were variations in the pattern of application during clinical practice. Among the ERAS protocols for CRS and HIPEC, pre and intra operative elements were the best-adhered components. Pre-admission rehabilitation, preoperative nutritional screening and care, multimodal prophylaxis for postoperative nausea and vomiting (PONV), DVT prophylaxis, maintenance of intraoperative normothermia, anaesthesia induction and ventilation, regional anaesthesia avoid use of opioids, early removal of urinary catheter, were well adopted. However, there were many practices followed by the respondents which would be considered to be in contradiction with the ERAS guidelines. Elective mechanical bowel preparation, pre-operative fasting protocol, cardiac output monitoring, goal-directed intravenous fluid administration were relatively less well-adopted.

Evidence and ERAS guidelines have supported the avoidance of routine mechanical bowel preparation and use only for left sided colonic resections, particularly due to adverse outcomes such as hypovolemia and dehydration and the fact that it does not decrease post-operative morbidity [6]. But nearly 62 % of the surgeons in the survey still insist on it routinely, probably because in CRS most patients need multi visceral resections and there is no way to predict to whom we perform only left sided resections [20].

Preoperative fasting of 2 h for clear fluids and 6 h for solid meals was followed by most surgeons, in accordance with the ERAS protocol. But few respondents still preferred overnight fasting, probably as per the guidelines issued by the Indian Society of Anaesthesiologists based on distinct eating habits of Indian population [21]. Oral consumption of complex carbohydrate, 2 h before induction of anaesthesia reduces the postoperative insulin resistance and improves postoperative outcomes [22]. Despite a strong recommendation in favour by ERAS protocol, only 26.2 % of the respondents reported to be following it. The poor implementation can be related to fear of aspiration. This highlights the need for customising the version of the ERAS protocol for the Indian population or educating the anaesthetists.

Surgeons agreed that the improvement in outcomes is not an effect of one particular element of the protocol, but rather an aggregation of marginal gains from all the elements. Even though it is not always possible to fully adhere to the protocol, as a whole they are proven to work, which is clearly confirmed in our analysis. The intervention which was most effective was intra operative goal directed therapy. Balanced fluid therapy as a single element lowers the morbidity rate, shortens LOS or decreases time to first flatus [23, 24].

Although there is a trend towards use of multimodal optimisation, the majority of the surgeons were still adhering to conventional post-operative care with variations in elements of ERAS guidelines. Abdominal and thoracic drains, Avoid use of antibiotic prophylaxis, early nasogastric tube removal, post-operative fluid overload monitoring and avoidance, initiation of early postoperative feeding and aggressive active mobilisation. However this practice has not been adopted in most of the surgeons. This could be attributed to many factors like pulmonary and cardiac comorbidities, extent of the surgery such as resection of the viscera (gastric/intestinal resection, Omentectomy, Diaphragmatic resection, splenectomy), amount of blood loss and duration of surgery.

The avoidance of post-operative fluid overload was not in practice among majority of respondents. The study demonstrates that certain traditional aspects of surgical care, such as the use of drains and nasogastric tubes and reliance on bowel sounds persists. Although routine NG decompression is not recommended [3]. In extensive upper abdominal procedure for CRS is like total supra colonic omentectomy, Lesser omentectomy, gastric resections or splenectomy is performed can lead to delayed gastric emptying necessitating post op NG drainage [25].

Guidelines have recommended against the routine use of peritoneal drains. Placement of drains can stimulate serous fluid production, and may lead to an increased risk of surgical-site infection and adhesions without any benefit of early detection of anastomotic leak [26, 27]. After CRS and HIPEC the possibility of intraperitoneal collections and abscess is higher and therefore most surgeons prefer placement of intraperitoneal drains.

65 % respondents in our survey stated the biggest barrier for implementation of ERAS was ensuring adherence and managing compliance. This is possibly because institutions without dedicated ERAS program have challenges in creating an effective ‘ERAS team’ (surgeon, anaesthetist, and nursing care givers), which is required for optimal implementation, adherence and compliance [28].

The main hindrances faced in the implementation of ERAS as per our survey respondents were safety issues, lack of level I evidence and administrative issues. While the majority of respondents’ attitudes were in favour of ERAS, more than half of respondents indicated that they felt that ERAS was associated with adverse outcomes and did not feel comfortable with few elements of the protocol. The main concerns was regarding NPO status, avoidance of NG tubes and drains and early initiation of regular diet. Although many studies have demonstrated safety, this has been with colorectal or gynaecological surgery where minimally invasive surgeries are very common. In CRS and HIPEC this is very different as the sheer extensive grade of surgery (peritonectomy and multivisceral resections) will lead to many physiological changes which makes few elements of guidelines not practical. This might be also be attributed to the fact of lack of studies of ERAS for CRS and HIPEC. There is also institutional variation in several perioperative practices, whereby hospitals already offering ERAS for other surgical specialities with well trained staff and co-ordinated effort from allied departments. One of the most important aspects is multidisciplinary and clinical support. The ERAS team includes pre-admission staff, dieticians, nurses, physiotherapists, social workers, occupational therapists and doctors. All team members must be familiar ERAS principles and be motivated and educated to carry out the program; they must be able to overcome traditional concepts, teaching and attitudes towards perioperative care. Increasing awareness among allied clinical departments, staff members of the hospital and demonstrating the cost benefits, patient satisfaction the may help in overcoming the administrative issues [29, 30].

There were some limitations to our study. First, the survey-based study represents clinicians’ opinions and may not represent real world practice. The respondents included only surgeons and did not include anaesthetists, intensive care specialists or nursing care providers. Due to the extensive nature of the ERAS protocol, all the components of ERAS could not be followed by the respondents.

## Conclusions

Our survey has shown that ERAS has have been adopted among most surgeon members of ISPSM, although certain aspects of perioperative practice have potential for improvement. The practice of ERAS should be encouraged in all. Conventional practices, traditional attitudes and non-intuitive protocols need to change and clinical staff have to adapt. Survey demonstrated that certain aspects of enhanced recovery are more commonly in practice by centres already following ERAS principles for other specialities. The dilemma is not only the implementation of ERAS protocols but also to improve the protocol and to achieve high compliance.

ERAS is a multidisciplinary concept, and a coordinated effort is needed between various specialities to improve penetration of the ERAS protocol in CRS and HIPEC. Training and formation of dedicated team is imperative. More robust clinical evidence is needed to convince the clinicians to include all elements of protocol to systematically incorporated into ERAS practices. Few aspects may be difficult to implement in all patients and more evidence is needed to recommend their routine use.
